# Five-year longitudinal study of frailty prevalence and course assessed using the Kihon Checklist among community-dwelling older adults in Japan

**DOI:** 10.1038/s41598-021-91979-6

**Published:** 2021-06-11

**Authors:** Masayuki Ohashi, Takuya Yoda, Norio Imai, Toshihide Fujii, Kei Watanabe, Hideki Tashi, Yohei Shibuya, Jin Watanabe, Naoto Endo

**Affiliations:** 1 Division of Comprehensive Musculoskeletal Medicine, Niigata University Graduate School of Medical and Dental Sciences, Niigata City, Japan; 2 Division of Orthopedic Surgery, Department of Regenerative and Transplant Medicine, Niigata University Graduate School of Medical and Dental Sciences, 1-757 Asahimachi Dori, Chuo-ku, Niigata City, 951-8510 Japan; 3 Division of Comprehensive Geriatrics in Community, Niigata University Graduate School of Medical and Dental Sciences, Niigata City, Japan; 4 Department of Orthopedic Surgery, Agano City Hospital, Agano City, Japan; 5 Division of Musculoskeletal Science for Frailty, Niigata University Graduate School of Medical and Dental Sciences, Niigata City, Japan

**Keywords:** Geriatrics, Risk factors

## Abstract

The aim of this study was to analyze the 5-year natural course of frailty status assessed with the Kihon Checklist (KCL) and the risk factors of transition towards frailty in community-dwelling older adults. We used the data from the postal KCL survey conducted by the municipal government between 2011 and 2016. The sample of the current study consisted of 551 older adults (265 men and 286 women) aged 65–70 years in 2011. The median KCL score increased from 2 (interquartile range 1–3) in 2011 to 3 (1–5) in 2016 (p < 0.001). Hence, the prevalence of frailty increased from 8.0 to 12.3% (p < 0.001). Regarding the 5-year transitions in frailty status, 68.3% of participants remained unchanged, while 21.4% transitioned towards a worse frailty status, and 10.3% towards an improved status. Of the 507 respondents who were robust or prefrail at the baseline, 44 experienced a transition towards frailty, indicating that the 5-year incidence of frailty was 8.7%. These 44 individuals had higher body mass indexes (BMI) and lower physical activity scores on the KCL than others (p < 0.05), the latter of which was an independent predictor of transition toward frailty in the multivariate analysis. This study was the first to evaluate the 5-year natural course of frailty status assessed using the KCL in community-dwelling elderly adults, in which the prevalence of frailty increased by 4.3%. To prevent transition towards frailty, maintaining optimal physical activity is recommended.

## Introduction

The population of older adults in Japan is growing rapidly, with the proportion of those aged 65 years or more at 27.7% in 2017^1^. Moreover, it is estimated to increase to 30% by 2025 and 37.7% by 2050^1^. This increasing number in Japan is expected to substantially contribute to healthcare costs. To prepare for the growing burden of an aging population, the Japanese government has started attempts to facilitate healthy aging by promoting the maintenance of their physical function, thereby preventing disability and dependence^2^, with frailty as a target.

Frailty is a common geriatric syndrome involving increased vulnerability to stressors or outcomes such as disability, requirement for long-term care, and death^3^. Frailty also includes vulnerability in psychological and social aspects of life^3^. Currently, several frailty criteria exist. One of the most widely used definitions of frailty is the frailty phenotype proposed by Fried et al. using data from the Cardiovascular Health Study^4^. On the other hand, Rockwood et al. released their accumulated deficits model of frailty, which considered not only the physical components of frailty, but also its psychosocial aspects^5^.

In Japan, the Kihon Checklist (KCL) was developed to identify community-dwelling older adults at risk of dependency^6, 7^. The KCL is a self-assessment tool comprising 25 yes/no items divided into seven dimensions including activities of daily living (ADL), physical activity, nutritional status, oral function, house-boundedness, cognitive status, and depressive mood (Table S1)^7^. It demonstrated good validity in identifying frailty defined with Fried’s criteria^8, 9^ and predicting the incidence of dependency and mortality within 3 years^10^. Moreover, it reportedly takes about 15 min for older adults to answer the KCL^11^. Therefore, annual health checkups using the KCL can be a cost- and time-effective method to maintain and improve the quality of life of older adults in a timely manner. Satake et al.^10^ performed a 3-year longitudinal study and demonstrated the relationships between the baseline KCL score and the incidence of dependency or death within 3 years. However, to our knowledge, there is no longitudinal data of the KCL scores per se in community-dwelling older adults. On the other hand, the age of 65–70 years has recently been regarded as the start of old age. Around this age range, people usually experience major life events, such as retirement, which generally has strong effects on physical activity and behavior^12, 13^. Therefore, people aged 65–70 years could be an important age group for preventing transitions towards frailty and focusing on older adults within this 5-year age range should reveal predictors for early interventions. Therefore, this study aimed to analyze the 5-year natural course of frailty status assessed with the KCL and the risk factors of transition towards frailty in community-dwelling older adults aged 65–70 years.

## Materials and methods

This study was a retrospective analysis of the postal KCL surveys conducted by the municipal government of Agano City, located in the northeast of Niigata Prefecture, Japan. The municipal government has annually conducted a postal KCL survey since 2011 for citizens aged 65 years and older as part of the long-term care prevention project. The government sent the self-administered questionnaire, the KCL, through the postal system, and the respondents sent it back to the government through mail after completing the questionnaire. The government collected the data of the KCL scores and the authors analyzed them. In 2011, Agano City had a population of 3013 people aged 65–70 years, of which 1053 older adults were randomly selected by the municipal government and surveyed. We excluded people who skipped any questions in the KCL from the current study. Out of the 1053 selected individuals, 774 completed the KCL in 2011. Moreover, 551 of them completed it in 2016. The current longitudinal study population consisted of the 551 older adults aged 65–70 years in 2011 who responded to the KCL surveys both in 2011 and in 2016 (follow-up rate, 52.3%).

### The Kihon Checklist (KCL)

The KCL is a self-reporting questionnaire consisting of 25 “yes” or “no” items, including 5 items for ADL, 5 for physical activity, 2 for nutritional status, 3 for oral function, 2 for house-boundedness, 3 for cognitive status, and 5 for depressed mood (Table S1)^7^. Each question was rated on a pass/fail basis, and the sum of all indices’ questions ranged from 0 to 25, with a higher KCL score indicating a higher risk for requiring support or care. In this study, according to the frailty criteria reported by Satake et al.^9^, a KCL score of 0 to 3 was considered as robustness, 4 to 7 as prefrailty, and 8 or higher as frailty. Transition of frailty status was classified into three categories based on changes between 2011 and 2016: improved, unchanged, and worsened transitions. In the improved transition, the total or dimensional scores of the KCL decreased in 2016 compared to those in 2011, while the score increased in the worsened transition. The 5-year incidence of frailty was calculated as a percentage of older adults who were frail in 2016 among those who were robust or prefrail in 2011. Moreover, older adults who were robust or prefrail in 2011 were divided into 2 groups according to their status in 2016; group A included older adults who were also robust or prefrail in 2016, and group B included those who were frail in 2016.

### Statistical analysis

Statistical analyses were performed using SPSS software (version 19; IBM Corp., Armonk, NY). Continuous data are expressed as mean ± standard deviation or median [interquartile range (IQR)], as appropriate. Differences in the KCL scores between 2011 and 2016 were evaluated using the Wilcoxon rank-sum test. Differences between groups A and B were analyzed using the unpaired t-test, the Mann–Whitney U test, and the chi-square test. A post hoc test for the chi-square test was performed using a residual analysis, if appropriate. Additionally, a stepwise logistic regression analysis was performed to identify independent risk factors for transition towards frailty, in which factors with p < 0.20 in the univariate analyses were included in the multivariate analysis. Correlation analysis was performed using Spearman’s rank correlation coefficient (rho). The strength of the correlation was determined as follows: weak (0.1–0.3), moderate (0.3–0.5), strong (0.5–1.0)^14^. The value of p < 0.05 was considered statistically significant. In the residual analysis, when the absolute value of the adjusted residual was greater than 1.96, the observed frequency was considered to be significantly different from the expected frequency at the level of p < 0.05.

### Ethics statement

This study was reviewed and approved by the institutional review board of Niigata University (2019-0221). All methods in this study were carried out in accordance with relevant guidelines and regulations. Informed consent was obtained from all participants.

## Results

**Figure 1:**
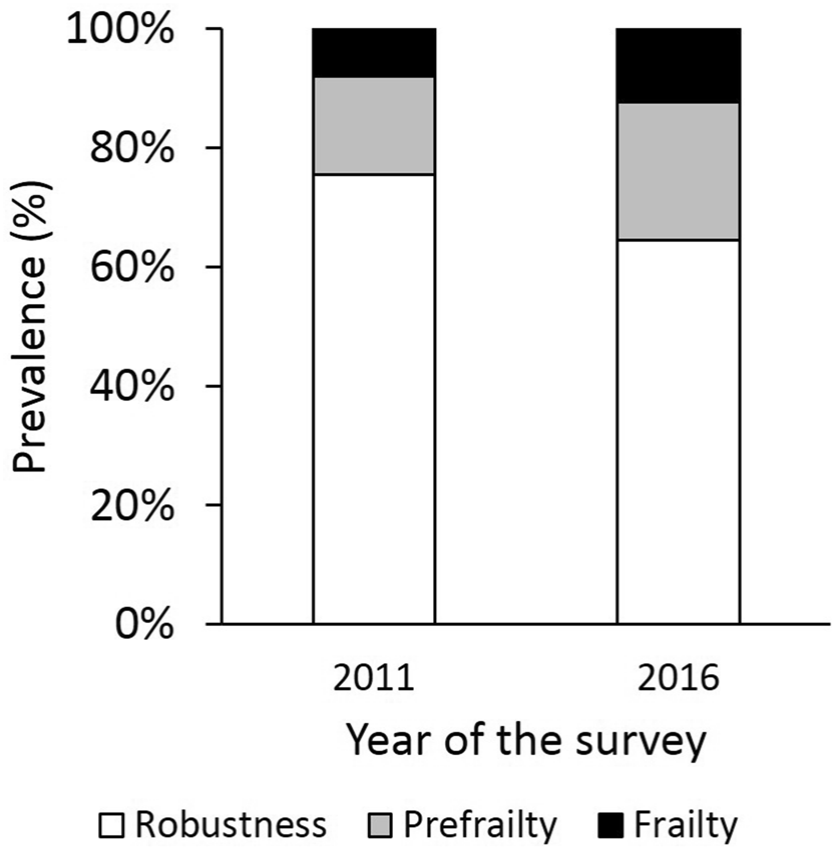
Each frailty status in 2011 and 2016.

Our cohort consisted of the 551 older adults including 265 men and 286 women, with the average age of 67.3 ± 1.5 years (range 65–70 years) in 2011. The median KCL score significantly increased from 2 (IQR 1–3) in 2011 to 3 (1–5) (p < 0.001). As a result, the prevalence of prefrailty and frailty significantly increase from 16.5% (n = 91) to 23.2% (n = 128) (adjusted residual, 2.793), and from 8.0% (n = 44) to 12.3% (n = 68) (adjusted residual, 2.393), while the prevalence of robustness significantly decreased from 75.5% (n = 416) to 64.4% (n = 355) (adjusted residual, − 4.008) (chi-square test, p < 0.001) (Fig. 1). On the other hand, body mass index (BMI), which was calculated using the answer to question 12 of the KCL (Table S1)^7^, did not change between 2011 [23.0 ± 3.2 kg/m^2^ (range, 14.5–34.7 kg/m^2^)] and 2016 [23.0 ± 3.3 kg/m^2^ (range 12.3–37.5 kg/m^2^)] (p = 0.10). According to the definition of adult overweight and obesity by Centers for Disease Control and Prevention^15^, the majority (n = 392, 71.1%) of older adults were normal weight in 2011, while 5.6% (n = 31) were underweight, 21.1% (n = 116) were overweight, and 2.2% (n = 12) were obese.

Table 1 shows transitions of seven dimensions in the KCL from 2011 to 2016. The incidences of transitions, which were improved, unchanged, and worsened transitions, significantly differ among dimensions (p < 0.001). Residual analyses demonstrated significantly higher incidences of worsened transitions in physical activity (28.9%) and oral function (25.1%), and significantly lower incidences of worsened transitions in nutritional status (15.8%), house-boundedness (14.0%), and cognitive status (16.9%) compared to the other dimensions (p < 0.05).

**Table 1:** Transitions of seven dimensions in the Kihon Checklist from the baseline to the 5-year follow-up.

	ADL	Physical activity	Nutritional status	Oral function	House-boundedness	Cognitive status	Depressive mood
Improved (cases, %)	64 (11.62%)	109 (19.78%)	49 (8.89%)	97 (17.60%)	59 (10.71%)	75 (13.61%)	83 (15.06%)
Unchanged (cases, %)	379 (68.78%)	283 (51.36%)	415 (75.32%)	316 (57.35%)	415 (75.32%)	383 (69.51%)	340 (61.71%)
Worsened (cases, %)	108 (19.60%)	159 (28.86%)	87 (15.79%)	138 (25.05%)	77 (13.97%)	93 (16.88%)	128 (23.23%)
Adjusted residuals for worsened transition	− 0.554	5.261*	− 2.948*	2.867*	− 4.088*	− 2.264*	1.727

*ADL* activities of daily living.

*Statistically significant at the level of p < 0.05.

**Figure 2:**
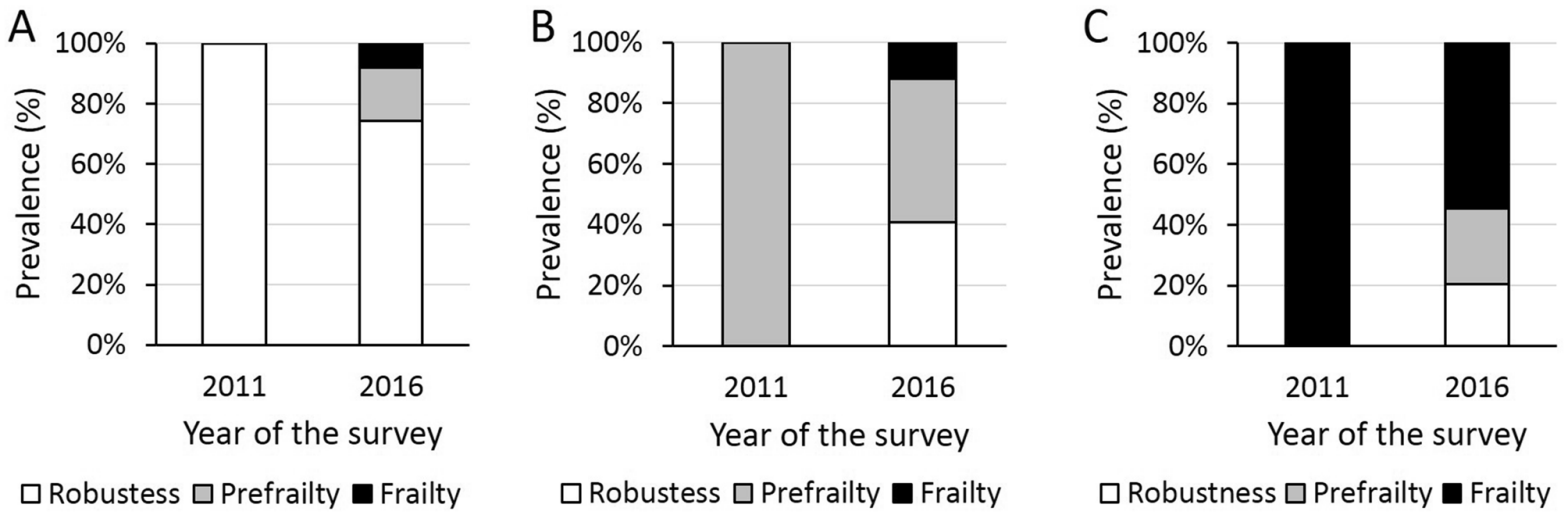
Transitions in frailty status by the status at the baseline. (**A**) Transitions in older adults who were robust in 2011. (**B**) Transitions in older adults who were prefrail in 2011. (**C**) Transitions in older adults who were frail in 2011.

Table 2 shows the prevalence of frailty status at the baseline (2011) and the 5-year follow-up survey (2016). The majority (68.3%) of older adults remained unchanged, while 21.4% transitioned towards a worsened frailty status in 5 years, and 10.3% towards an improved status. Among older adults who were robust at the baseline, 25.7% demonstrated a transition towards a worsened frailty status, in which 17.8% was prefrailty, and 7.9% was frailty (Fig. 2A). On the other hand, 40.7% of older adults who were prefrail at the baseline transitioned towards robustness in 5 years, while 12.1% experienced a transition towards frailty (Fig. 2B). Interestingly, approximately half of older adults who were frail at the baseline improved their frailty status, transitioning towards prefrailty (25.0%) or robustness (20.5%) (Fig. 2C).

**Table 2:** Transitions in frailty status from the baseline to the 5-year follow-up.

Baseline status (2011)	Status at 5-year follow-up (2016)		
	Robust	Prefrailty	Frailty
Robust	309 (56.1%)	74 (13.4%)	33 (6.0%)
Prefrailty	37 (6.7%)	43 (7.8%)	11 (2.0%)
Frailty	9 (1.6%)	11 (2.0%)	24 (4.4%)

Data are shown as the number of cases (prevalence, %).

At the baseline, 507 older adults had non-frailty status (robust or prefrailty). Of these, 44 experienced a transition towards frailty (group B), indicating that the 5-year incidence of frailty was 8.7%. In the comparison of the baseline data between groups A and B (Table 3), BMI (mean; 22.9 kg/m^2^ in group A, 24.5 kg/m^2^ in group B, p = 0.035), and physical activity score of the KCL (median; 0 in group A, 1 in group B, p = 0.002) were significantly higher in group B than A. In a multivariate analysis, physical activity score of the KCL (odds ratio, 1.6; 95% confidence interval, 1.1–2.2; p = 0.009) independently predicted transition towards frailty (Table 4). Moreover, we found a positive correlation between BMI and physical activity score (rho = 0.227, p = 0.021). On the other hand, there were no significant differences in age, sex, and the other dimensional scores of the KCL between groups A and B.

**Table 3:** Comparisons of the baseline data between the older adults who experienced a transition from non-frailty to frailty and those who did not.

Baseline data (2011)	Group A (n = 463)	Group B (n = 44)	p-value
Age (years, mean ± SD)	67.3 ± 1.5	67.4 ± 1.5	0.63
**Sex (cases)**			
Male	227	20	
Female	236	24	0.65
BMI (kg/m^2^, mean ± SD)	22.9 ± 3.0	24.5 ± 4.6	0.035*
**KCL score [median (IQR)]**			
Activities of daily living	0 (0)	0 (0–1)	0.19
Physical activity	0 (0–1)	1 (0–1)	0.002*
Nutritional state	0 (0)	0 (0)	0.53
Oral function	0 (0–1)	0 (0–1)	0.13
Houseboundness	0 (0)	0 (0)	0.12
Cognitive function	0 (0)	0 (0)	0.97
Depressive mood	0 (0)	0 (0)	0.49

Group A included older adults who were non-frail both in 2011 and 2016 and group B included those who had transitions from non-frailty to frailty.

*BMI* body mass index, *SD* standard deviation, *KCL* Kihon Checklist, *IQR* interquartile range.

*p < 0.05.

**Table 4:** Multiple logistic regression analysis of risk factors at the baseline for transition towards frailty.

	Odds ratio (95% CI)	p-value
BMI	–	0.47
**KCL score**		
ADL	–	0.28
Physical activity	1.6 (1.1–2.2)	0.009*
Oral function	–	0.21
Houseboundness	–	0.09

*BMI* body mass index, *KCL* Kihon Checklist, *ADL* activity of daily living, *CI* confidence interval.

*p < 0.05.

## Discussion

We evaluated the frailty status assessed using the KCL and found that in community-dwelling older adults aged 65–70 years, 75.5% had a robust status, 16.5% had prefrailty, and 8.0% had frailty.

Regarding the previously reported prevalence of frailty among community-dwelling older adults, Choi et al. reviewed 6 national population-based surveys and reported that the prevalence of frailty defined using Fried’s criteria demonstrated significant differences among countries, ranging from 5.8 to 27.3%^15^. In the previous systematic review, age-stratified prevalence of frailty was approximately 4% in people aged 65–69 years^16^. Among the older adults in Japan, the prevalence of frailty defined with Fried’s criteria reportedly ranged from 1.5 to 11.6%^9, 17–20^, while that defined using the KCL ranged from 4 to 17.2%^10, 17, 21^. On the other hand, the age-stratified prevalence of frailty defined with Fried’s criteria was reported to be 1.9–5.4% for Japanese people aged 65–69 years^19, 20^, and 4.0% for those aged 65–74 years^18^. Considering that the prevalence of frailty evaluated with the KCL was reported to be slightly higher than that using Fried’s criteria^17^, we believe that the baseline frailty status of the current study was similar to that of the previously reported cohorts.

Recently, the KCL has been reported to be a predictor of long-term care risk^9^, healthy life expectancy^22^, and cognitive function^23^. Therefore, in Japan, a rapidly aging society, the KCL could be a useful tool to monitor and evaluate frailty status at a low cost because the survey using the KCL is performed with a self-reporting questionnaire through mail. On the other hand, to design appropriate frailty interventions and identify optimal target populations to prevent or delay progression, it is imperative to understand the progressive course of frailty and to predict how the frailty status of older people evolves over time. However, little is known about the natural course of frailty status defined using the KCL.

In the current study, we found that the total score of the KCL significantly increased from the baseline to the 5-year follow-up and the prevalence of frailty defined using the KCL significantly increased from 8.0 to 12.3%. These results are compatible with previous studies, which reported that advanced age is a strong risk factor for frailty status^20, 21^. Regarding transitions in frailty status defined using Fried’s criteria, Kojima et al. reviewed 16 studies and reported that 13.7% of older adults improved, 29.1% worsened, and 56.5% remained unchanged over a mean of 3.9 years^24^. Our study evaluated frailty status as assessed with the KCL and demonstrated that 11.6% of older adults improved, 19.6% worsened, and 68.8% maintained the same frailty status in 5 years, in which the rate of worsened transition was slightly lower than that reported in the previous meta-analysis^24^. It might be due to the difference of criteria for defining frailty status, which could affect the prevalence of frailty^17^. Moreover, it might also be due to the younger age of our participants (mean age 67.3 years, range 65–70 years) than that in the previous meta-analysis, in which most studies had participants with a mean age older than 70 years^24^. Indeed, previous studies showed that older people’s frailty status is likely to worsen^25–27^. On the other hand, it should be noted that frailty is not an irreversible status, but a reversible and dynamic status involving improvement as well as progression. Actually, in our study, 45.5% of frail elderly individuals improved their status to prefrailty or robustness. This reversibility of frailty status indicates that it is worth the effort to attempt to establish appropriate interventions for frailty. For this aim, it is necessary to clarify the risk factors of a transition towards frailty, and thus, we performed comparisons of the baseline data between the older adults who experienced a transition towards frailty (group B) and those who did not (group A) followed by the multiple logistic regression analysis. In the univariate analysis, BMI was significantly higher in group B (mean, 24.5 kg/m^2^) than in group A (mean, 22.9 kg/m^2^). This is in line with the results of the review article in which obesity and high waist circumference demonstrated highly convincing results for an association with frailty^28^. Additionally, the physical activity score of the KCL was significantly higher in group B (median, 1) than group A (median, 0), indicating that those in group B had a significantly worsened status in the physical activity category than those in group A. Moreover, multiple logistic regression analysis demonstrated that the worsened status in the physical activity was an independent predictor of the risk factor of transition towards frailty in 5 years. We also demonstrated a weak but statistically significant positive association between BMI and physical activity score, which means that the greater the BMI, the lesser the physical activity. Therefore, although it could be likened to the chicken or the egg debate regarding whether the increase in BMI or the decrease in physical activity comes first, we believe that improving physical activity primary, and as a result, maintaining proper BMI as well, for the prevention of transition towards frailty should be recommended to older adults with a physical activity score of 1 or higher. On the other hand, Ho et al. reported that Asian individuals in midlife with lower than normal weight, as well as those who were obese, had an increased risk of developing frailty in 8 years^29^. Moreover, Nishida et al. reported that weight loss over 2 years was associated with a higher death rate and the need for long-term care insurance during the subsequent 3-year follow-up period among older Japanese participants^30^. Therefore, not only obesity but also lower than normal weight should be avoided and the optimal weight-targeting strategy might be appropriate for community-dwelling older adults. Because our cohort included only 5.6% of older adults with underweight, we might not be able to find any negative impacts of underweight on frailty status.

Limitations exist in the current study. First, this is a retrospective analysis of the questionnaire surveys conducted by the government; therefore, we could analyze 5-year follow-up data only in older adults aged 65–70 years due to the method of selecting participants of the postal KCL survey conducted by the government. However, we believe that it reduced heterogeneity with age, which is one of the strongest factors for frailty. Secondly, we had no data on medical history, physical and social activity, and lifestyle. Moreover, we did not measure the frailty status with any physical tests. Therefore, the cohort of this study might include people who had already had disability or cognitive dysfunction at the baseline. Thirdly, it was unknown the reasons for unanswered survey, which might be because those people had died or had suffered some kind of disability that prevent them from answering in the second survey. Fourthly, we could not know how participants measured their body weight including equipment used, whether with or without clothes, or the season of the year. Fifthly, although the three status, which were robust, prefrailty, and frailty, were adopted, we performed two group comparisons using non-frail and frail status to analyze the risk for frailty transition due to the relatively small sample size of this study. Finally, the “optimal” exercise regimen and BMI to prevent frailty are still unclear. To address these limitations, further prospective, comprehensive studies that include older adults aged over 70 years is necessary for future research.

In conclusion, to our knowledge, this study is the first to evaluate frailty status and its longitudinal transitions using the KCL, which is a promising tool to easily and cost-effectively evaluate frailty status. We demonstrated that 8.0% of community-dwelling older adults aged 65–70 years were frail. Regarding transitions in frailty status, 10.3% of older adults improved, 21.4% worsened, and 68.2% maintained their status over the course of 5 years. The 5-year incidence of frailty status was 8.7%. To prevent a transition towards frailty, maintaining optimal levels of physical activity should be recommended.

## Supplementary Information

Supplementary Information.
