# Supplementary material for: Five-year longitudinal study of frailty prevalence and course assessed using the Kihon Checklist among community-dwelling older adults in Japan
Source: Sci Rep. 2021 Jun 11;11:12399. doi: 10.1038/s41598-021-91979-6 (PMC8196037; doi:10.1038/s41598-021-91979-6)
Supplement: Supplementary file 1 — Supplementary Information. [file 41598_2021_91979_MOESM1_ESM.docx]

**Five-year longitudinal study of frailty prevalence and course assessed using the Kihon Checklist among community-dwelling older adults in Japan**

Masayuki Ohashi^1,2*^, Takuya Yoda^1^, Norio Imai^1,3^, Toshihide Fujii^4^, Kei Watanabe^2^, Hideki Tashi^5^, Yohei Shibuya^2^, Jin Watanabe^2^, Naoto Endo^2^

1. Division of Comprehensive Musculoskeletal Medicine, Niigata University Graduate School of Medical and Dental Sciences, Niigata City, Japan

2. Division of Orthopedic Surgery, Department of Regenerative and Transplant Medicine, Niigata University Graduate School of Medical and Dental Sciences, Niigata City, Japan

3. Division of Comprehensive Geriatrics in Community, Niigata University Graduate School of Medical and Dental Sciences, Niigata City, Japan

4. Department of Orthopedic Surgery, Agano City Hospital, Agano City, Japan

5. Division of Musculoskeletal Science for Frailty, Niigata University Graduate School of Medical and Dental Sciences, Niigata City, Japan

**Supporting information**

**Table S1.** Kihon Checklist^7^

| 1. | Do you go out by bus or train by yourself? | 0. Yes | 1. No |
| --- | --- | --- | --- |
| 2. | Do you go shopping to buy daily necessities by yourself? | 0. Yes | 1. No |
| 3. | Do you manage your own deposits and saving at the bank? | 0. Yes | 1. No |
| 4. | Do you sometimes visit your friends? | 0. Yes | 1. No |
| 5. | Do you turn to your family or friends for advice? | 0. Yes | 1. No |
| 6. | Do you normally climb stairs without using handrails or wall for support? | 0. Yes | 1. No |
| 7. | Do you normally stand up from a chair without any aids? | 0. Yes | 1. No |
| 8. | Do you normally walk continuously for 15 minutes? | 0. Yes | 1. No |
| 9. | Have you experienced a fall in the past year? | 1. Yes | 0. No |
| 10. | Do you have a fear of falling while walking? | 1. Yes | 0. No |
| 11. | Have you lost 2kg or more in the past 6 months? | 1. Yes | 0. No |
| 12. | Height: cm, weight: kg, BMI†: kg/m^2^ If BMI is less than 18.5, this item is scored | 1. Yes | 0. No |
| 13. | Do you have any difficulties eating tough foods compared to 6 months ago? | 1. Yes | 0. No |
| 14. | Have you choked on your tea or soup recently? | 1. Yes | 0. No |
| 15. | Do you often experience having a dry mouth? | 1. Yes | 0. No |
| 16. | Do you go out at least once a week? | 0. Yes | 1. No |
| 17. | Do you go out less frequently compared to last year? | 1. Yes | 0. No |
| 18. | Do your family or your friends point out your memory loss? E.g. "You always ask the same question over and over again"? | 1. Yes | 0. No |
| 19. | Do you make a call by looking up phone numbers? | 0. Yes | 1. No |
| 20. | Do you find yourself not knowing today's date? | 1. Yes | 0. No |
| 21. | In the last two weeks have you felt lack of fulfilment in your daily life? | 1. Yes | 0. No |
| 22. | In the last two weeks have you felt a lack of joy when doing the things you used to enjoy? | 1. Yes | 0. No |
| 23. | In the last two weeks have you felt difficulty in doing what you could do easily before? | 1. Yes | 0. No |
| 24. | In the last two weeks have you felt helpless? | 1. Yes | 0. No |
| 25. | In the last two weeks have you felt tired without a reason? | 1. Yes | 0. No |

^†^BMI, body mass index.
